# Proteomic Analysis of Embryogenesis and the Acquisition of Seed Dormancy in Norway Maple (*Acer platanoides* L.)

**DOI:** 10.3390/ijms150610868

**Published:** 2014-06-17

**Authors:** Aleksandra Maria Staszak, Tomasz Andrzej Pawłowski

**Affiliations:** Institute of Dendrology Polish Academy of Sciences, Parkowa 5, 62-035 Kórnik, Poland; E-Mail: staszak.a@gmail.com

**Keywords:** morphogenesis, physiological dormancy, seed development, seed maturation, trees

## Abstract

The proteome of zygotic embryos of *Acer platanoides* L. was analyzed via high-resolution 2D-SDS-PAGE and MS/MS in order to: (1) identify significant physiological processes associated with embryo development; and (2) identify changes in the proteome of the embryo associated with the acquisition of seed dormancy. Seventeen spots were identified as associated with morphogenesis at 10 to 13 weeks after flowering (WAF). Thirty-three spots were associated with maturation of the embryo at 14 to 22 WAF. The greatest changes in protein abundance occurred at 22 WAF, when seeds become fully mature. Overall, the stage of morphogenesis was characterized by changes in the abundance of proteins (tubulins and actin) associated with the growth and development of the embryo. Enzymes related to energy supply were especially elevated, most likely due to the energy demand associated with rapid growth and cell division. The stage of maturation is crucial to the establishment of seed dormancy and is associated with a higher abundance of proteins involved in genetic information processing, energy and carbon metabolism and cellular and antioxidant processes. Results indicated that a glycine-rich RNA-binding protein and proteasome proteins may be directly involved in dormancy acquisition control, and future studies are warranted to verify this association.

## 1. Introduction

Seeds are formed during the reproductive stage of higher plants. Embryogenesis occurs after fertilization of the egg and is characterized in dicot plants by different stages of morphogenesis and physiological maturation. Embryo morphogenesis is characterized by cell division and differentiation. During maturation, seeds undergo changes that terminate further development and growth of the embryo, prepare the seed for desiccation (drying), preserve cellular components required for continued metabolism following desiccation, ready the seeds for germination and, in some species, impose a dormant state [1]. Dormancy is characterized by the failure of a seed to complete germination under favorable conditions. It is an innate seed property that defines the conditions that will allow a seed to germinate. Seed dormancy prevents germination in environments that are unfavorable for subsequent plant growth and reproduction. Dormancy allows for the dispersal of seeds in space and time [2]. Breaking of seed dormancy and subsequent germination are governed by environmental cues, including temperature, light, nitrate and some other factors [1].

According to the classification of Baskin and Baskin [3], five major types of dormancy can be defined, including physiological dormancy (PD). PD is a characteristic of fully-developed, mature embryos. It is determined by physiological factors located in the embryo and/or surrounding tissue layers (endosperm, testa) [4]. Considerable research has used a molecular approach, based on model plants, such as *Arabidopsis*, and cereals to study PD, but seeds of these species do not exhibit a deep PD [4]. In contrast, seeds of many temperate tree species are characterized by a strong and deep physiological dormancy localized in the mature embryo [5]. Embryos excised from these seeds either do not grow or produce abnormal seedlings [2]. Seed dormancy is also associated to some degree with seed viability, which directly impacts the ability to preserve and store seeds [6].

Since most of the knowledge pertaining to seed dormancy is based on studies in *Arabidopsis* seeds (characterized by coat-imposed dormancy) [7], investigations of seeds from species that exhibit embryo dormancy can provide new insights into the mechanism of dormancy. Norway maple (*A. platanoides* L.) is a temperate zone tree species whose seed exhibits a deep, embryo-based, physiological dormancy. It produces fruit, called a samara, which contains a seed without endosperm within a thin brown seed coat. The cotyledons inside the seed are already green prior to germination, when the seed is still completely dormant. Samaras are shed prior to winter [8], before seeds develop desiccation tolerance and a state of deep dormancy. Seeds require cold stratification (3 °C) for approximately fifteen weeks to break dormancy and to be capable of germinating [9].

Proteomic technologies provide a great opportunity to investigate broad aspects of developmental biology, including plant reproduction [9,10]. Proteomic studies provide robust data about the relationship between biological function, as represented by the presence and abundance of specific proteins, and physiological changes [11]. Proteomics is a powerful tool for functional analysis [12] and is now being used in studies on seed development and dormancy [13,14]. Proteomic analyses of seed development have been performed on species that are characterized by either a lack of or weak dormancy, including legumes [15,16], *Arabidopsis* [17] and rapeseed [18,19]. The most highly-identified proteins have been those related to central metabolism, followed by those related to cellular structure, as well as many unknown proteins [20]. *Acer platanoides* is the model system for investigating broad aspects of the physiology, biochemistry and molecular biology of tree seed development, storage and germination, including unique deep physiological embryo dormancy [11]. For that reason, it was chosen as the object of investigation.

The main objective of the present study was to identify proteins associated with and, therefore, potentially regulating embryo development in seeds of Norway maple (*Acer platanoides* L.). Correlation studies between the protein patterns and physiological characteristics of seeds would help to decipher the proteins associated with the deep physiological dormancy acquisition, seed growth and desiccation tolerance acquisition. The study focused mainly on the acquisition of deep physiological dormancy. Samples for proteomic analysis were collected during two stages of seed development, embryo morphogenesis and embryo maturation. Results are discussed using a systems biology approach [21], based upon the obtained data, information from public databases and the existing literature.

## 2. Results and Discussion

### 2.1. Proteomic Analysis of Embryo Morphogenesis

**Figure 1:**
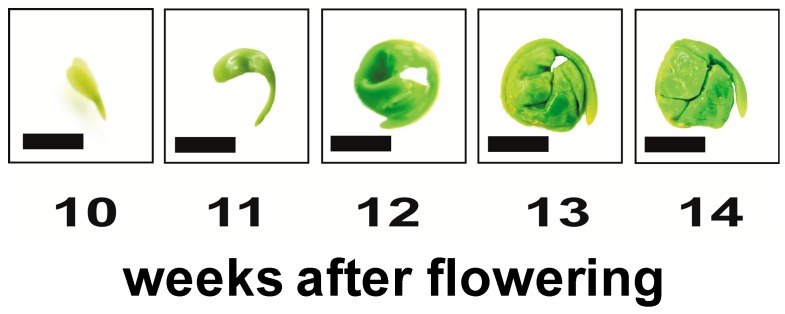
Embryogenesis of *A. platanoides* L. from 10 through 14 weeks after flowering (WAF). Bar = 5 mm.

**Figure 2:**
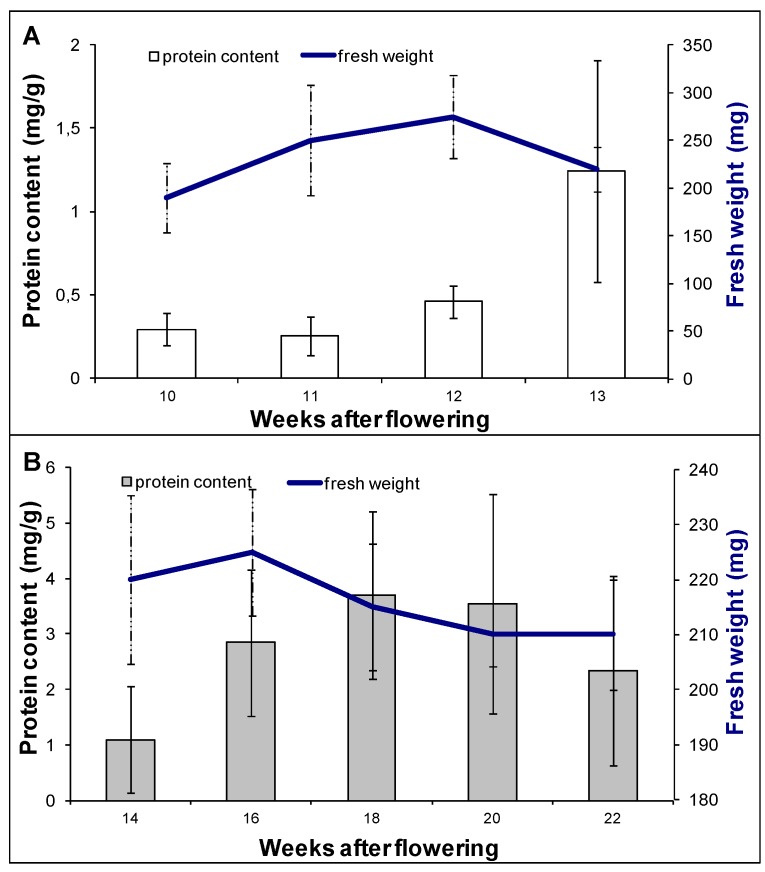
Protein content (columns) and fresh weight (line) of embryos of *A. platanoides* L. during morphogenesis: (**A**) 10 through 13 weeks after flowering (WAF) and maturation; (**B**) 14–22 WAF. Error bars represent SD (*n* = 3).

The morphological stage of Norway maple embryo development was monitored between 10 and 13 weeks after flowering (WAF) (Figure 1). At 10 WAF, embryos were vertical, and the cotyledons and root axes were recognizable. During the next three weeks, the embryo axes and, especially, the cotyledons enlarged in size. At 13 WAF, morphogenetic changes were complete, and the embryos could be classified as morphologically mature. Embryo fresh weight was also measured when samples were collected (Figure 2A). An increase in fresh weight was observed from 10 to 12 WAF followed by a decrease in week 13. An increase in protein content was also observed over the same time period (Figure 2A).

**Figure 3:**
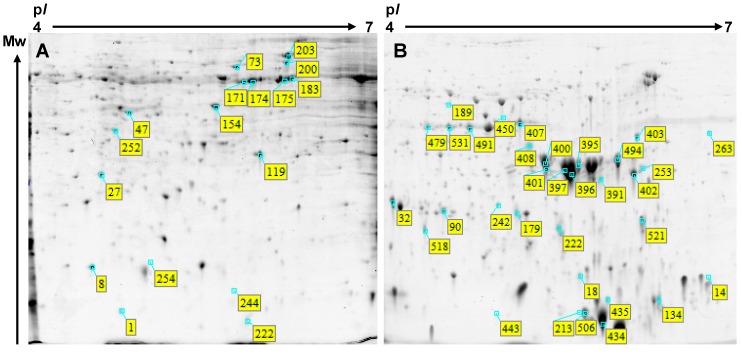
Representative images showing spot identification and localization of proteins extracted from embryos of *A. platanoides* during morphogenesis (**A**) and maturation (**B**). Proteins that varied in abundance during the period of embryo morphogenesis (10–13 weeks after flowering, WAF) and embryo maturation (14–22 WAF) are indicated by the yellow boxes and blue indicators. Complete information on these proteins is provided in Table 1, Table 2 and Table S1.

To identify proteins associated with this period of morphogenetic development, two-dimensional electrophoresis (2-DE) patterns of proteins from samples collected weekly from 10 to 13 WAF were analyzed in three biological replicates. An average of 349 Coomassie Blue stained spots were detected on each gel using Image Master 7 Platinum software. A total of 17 spots, representing approximately 5% of the total number of spots on a master gel (Figure 3A), exhibited significant changes in volume (*i.e.*, abundance) as determined by analysis of variance (ANOVA) and Tukey-Kramer HSD test (JMP software, SAS Institute, Cary, CA, USA) and were identified by mass spectrometry (MS). Due to the inability to match proteins to a species-specific database (a lack of the *A. platanoides* annotated genome sequence), proteins were identified using the entire Viridiplantae section of the NCBInr database (National Centre for Biotechnology Information, Bethesda, MD, USA).

**Table 1:** Identification of proteins in *Acer platanoides* that significantly changed in abundance during the period of embryo morphogenesis. For each spot, values for nominal (computed) and observed p*I*s and *M*_r_ and the mean normalized volume of each of the analyzed variants are indicated. Spots were subjected to analysis of variance (ANOVA) and the Tukey-Kramer HSD test (JMP software, SAS Institute, Cary, CA, USA) to select spots that significantly varied (*p* < 0.05) in abundance during morphogenesis.

Spot No. ^a^	Assignment	Species	gi No.	Mean % Volume ^b^ Weeks After Flowering (WAF)				Observed *M*_r_/p*I*	Nominal *M*_r_/p*I*	Score	Cov. (%) ^c^	Pept ^d^	Mean % Volume Graph
				10	11	12	13						
*Cellular processes*													
8	copper/zinc-superoxide dismutase	*Litchi* *chinensis*	gi|164654158	1.08 ± 0.09 ^b^	0.76 ± 0.17 ^b^	2.19 ± 0.34 ^a^	1.06 ± 0.31 ^b^	18/6.33	15/5.47	483	21	15	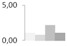
154	actin	*Boehmeria* *nivea*	gi|255115691	1.69 ± 0.23 ^a^	0.86 ± 0.45 ^a^^,b^	0.37 ± 0.45 ^b^	0.42 ± 0.19 ^b^	53/5.4	42/5.31	3161	69	83	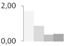
171	tubulin α chain	*Ricinus* *communis*	gi|255552898	1.40 ± 0.11 ^a^	0.71 ± 0.17 ^b^	0 ^c^	0.56 ± 0.21 ^b^	63/5.19	50/4.96	3172	47	65	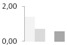
174	α tubulin	*Betula* *pendula*	gi|214003725	2.27 ± 0.04 ^a^	1.78 ± 0.11 ^b^	0 ^d^	0.58 ± 0.20 ^c^	63/5.13	50/4.93	7151	59	175	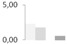
175	tubulin β-7 chain	*Zea mays*	gi|162460038	1.06 ± 0.02 ^a^	0.81 ± 0.18 ^a^	0 ^b^	0.12 ± 0.21 ^b^	64/4.88	50/4.72	1625	43	53	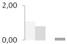
183	tubulin β chain	*Ricinus* *communis*	gi|255558856	1.43 ± 0.30 ^a^	1.01 ± 0.27 ^a^	0 ^b^	0 ^b^	65/4.82	62/4.77	3946	46	132	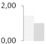
*Genetic information processing*													
1	nonspecific lipid transfer protein	*Vitis vinifera*	gi|359486799	0.96 ± 0.45 ^b^	0.50 ± 0.22 ^b^	2.03 ± 0.21 ^a^	0.87 ± 0.33 ^b^	14/6.11	13/9.20	225/84	9	1	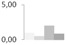
73	RuBisCO large subunit-binding protein subunit β, chloroplastic	*Pisum* *sativum*	gi|2506277	1.08 ± 0.06 ^a^	0.55 ± 0.21 ^a^^,b^	0.26 ± 0.45 ^b^	0.36 ± 0.16 ^b^	71/5.24	63/6.05	6509	47	161	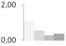
200	RuBisCO large subunit-binding protein subunit α, chloroplastic	*Brassica* *napus*	gi|1351030	0.98 ± 0.24 ^a^^,b^	0.61 ± 0.03 ^a^^,b^	0 ^b^	3.03 ± 1.88 ^a^	74/4.87	57/4.84	6947	22	167	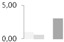
*Metabolism*													
27	triosephosphate isomerase, cytosolic	*Coptis* *japonica*	*gi|136057*	0.37 ± 0.21 ^b^	0.82 ± 0.26 ^a^^,b^	0.96 ± 0.18 ^b^	0.48 ± 0.12 ^a^^,b^	33/6.26	27/5.54	1986	16	48	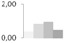
47	α-1,4-glucan-protein synthase (UDP-forming)	*Ricinus* *communis*	gi|255547137	0.70 ± 0.14 ^a^^,b^	1.19 ± 0.13 ^a^	0.80 ± 0.33 ^a^^,b^	0.26 ± 0.23 ^b^	50/6.05	41/5.82	1259	29	39	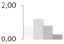
119	oxygen evolving enhancer protein 1	*Litchi* *chinensis*	gi|326467059	0.77 ± 0.04 ^a^^,b^	1.52 ± 0.55 ^a^	0.33 ± 0.57 ^b^	0.97 ± 0.36 ^a^^,b^	38/5.7	35/5.86	1660	30	52	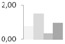
222	cytochrome b6-f complex iron-sulfur subunit, chloroplastic	*Pisum* *sativum*	gi|136707	0.79 ± 0.30 ^b^	1.33 ± 0.81 ^b^	5.51 ± 4.85 ^a^	6.98 ± 4.13 ^a^	13/5.16	24/8.63	108	5	2	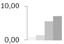
244	triosephosphate isomerase, cytosolic	*Zea mays*	gi|226495391	0 ^b^	0.13 ± 0.01 ^b^	4.41 ± 3.83 ^a^^,b^	9.27 ± 4.76 ^a^	16/5.26	27/5.52	128	10	2	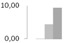
252	fructose-bisphosphate aldolase, putative	*Ricinus* *communis*	gi|255581400	0.48 ± 0.14 ^a^	0.31 ± 0.09 ^a^^,b^	0.06 ± 0.11 ^b^	0.21 ± 0.21 ^a^^,b^	44/6.16	42/6.78	1362	24	42	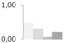
*Unclassified*													
203	protein disulfide-isomerase	*Vitis vinifera*	*gi|225459587*	1.76 ± 0.08 ^a^	2.61 ± 0.86 ^a^	0 ^b^	0 ^b^	78/4.85	56/4.93	238	11	8	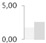
254	unknown	*Populus/italic>* *trichocarpa*	gi|118484746	0 ^b^	0 ^b^	0 ^b^	2.27 ± 0.93 ^a^	18/5.90	41/6.5	93	4	1	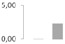

^a^ The spot number is the index in the reference gel; ^b^ The mean value with the standard deviation of three spot volumes at each analyzed stage: 10, 11, 12 and 13 WAF. Levels not connected by the same letter are significantly different; ^c^ Protein sequence coverage; ^d^ Number of peptide sequences.

**Table 2:** Identification of proteins in *Acer platanoides* that significantly changed in abundance during the period of embryo maturation. For each spot, values for nominal (computed) and observed p*I*s and *M*_r_ and the mean normalized volume of each of the analyzed variants are indicated. Spots were subjected to ANOVA and the Tukey-Kramer HSD test (SAS Institute) to select spots that significantly varied (*p* < 0.05) in abundance during maturation.

Spot No. ^a^	Assignment	Species	gi No.	Mean % Volume ^b^ WAF					Nominal *M*_r_/p*I*	Observed *M*_r_/p*I*	Score	Cov. (%) ^c^	Pept ^d^	Mean % Volume Graph
				14	16	18	20	22						
*Metabolism*														
32	glutathione S-transferase	*Catharanthus* *roseus*	gi|67973220	0.40 ± 0.15 ^a^^,b^	0.15 ± 0.18 ^b^	0.25 ± 0.12 ^b^	0.31 ± 0.28 ^a^^,b^	0.78 ± 0.11 ^a^	24/5.6	32/6.83	499	3	22	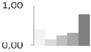
189	monodehydroascorbate reductase, chloroplastic	*Vitis vinifera*	gi|359474156	0.00 ± 0.00 ^b^	0.06 ± 0.01 ^a^	0.07 ± 0.01 ^a^	0.07 ± 0.02 ^a^	0.08 ± 0.02 ^a^	54/8.34	61/6.46	1099	14	27	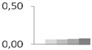
222	triosephosphate isomerase	*Gossypium* *hirsutum*	gi|295687231	0.01 ± 0.02 ^b^	0.01 ± 0.02 ^b^	0.06 ± 0.07 ^b^	0.17 ± 0.03 ^a^^,b^	0.36 ± 0.15 ^a^	33/6.66	26/5.58	253	16	5	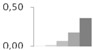
242	peptide methionine sulfoxide reductase-like	*Vitis vinifera*	gi|225454994	0.01 ± 0.01 ^c^	0.05 ± 0.02 ^b^^,c^	0.07 ± 0.01 ^a^^,b^	0.10 ± 0.01 ^a^^,b^	0.13 ± 0.04 ^a^	28/8.65	31/6.02	158	17	6	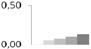
263	glyceraldehyde-3-phosphate dehydrogenase, cytosolic	*Taxus baccata*	gi|3023813	0.00 ± 0.01 ^c^	0.02 ± 0.01 ^c^	0.03 ± 0.01 ^b^^,c^	0.07 ± 0.02 ^a^^,b^	0.09 ± 0.02 ^a^	37/6.41	52/4.51	120	10	4	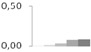
391	lactoylglutathione lyase	*Vitis vinifera*	gi|359483362	0.07 ± 0.02 ^b^	0.22 ± 0.14 ^a^^,b^	0.24 ± 0.05 ^a^	0.25 ± 0.02 ^a^	0.26 ± 0.01 ^a^	33/5.5	40/5.28	288	17	6	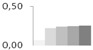
400	ascorbate peroxidase	*Retama raetam*	gi|24496465	1.10 ± 0.22 ^c^	1.78 ± 0.41 ^b^^,c^	2.01 ± 0.24 ^a^^,b^	2.62 ± 0.34 ^a^	2.16 ± 0.18 ^a^^,b^	39/7.03	45/5.68	229	11	4	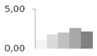
401	succinate semialdehyde dehydrogenase, putative	*Ricinus* *communis*	gi|255577875	0.43 ± 0.03 ^b^	0.51 ± 0.23 ^b^	0.45 ± 0.09 ^b^	0.97 ± 0.07 ^a^	0.86 ± 0.27 ^a^^,b^	66/8.84	44/5.67	174	6	3	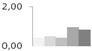
402	enolase	*Spinacia* *oleracea*	gi|8919731	0.36 ± 0.02 ^c^	0.64 ± 0.10 ^b^	0.86 ± 0.02 ^b^	1.22 ± 0.19 ^a^	1.37 ± 0.08 ^a^	48/5.49	41/5.04	381	16	6	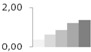
407	enolase	*Alnus* *glutinosa*	gi|3023685	0.28 ± 0.07 ^b^	0.58 ± 0.24 ^a^^,b^	1.04 ± 0.34 ^a^	0.83 ± 0.25 ^a^^,b^	0.77 ± 0.28 ^a^^,b^	48/5.41	55/5.86	392	16	6	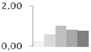
408	proline iminopeptidase	*Vitis vinifera*	gi|359475506	0.01 ± 0.02 ^b^	0.28 ± 0.03 ^a^	0.30 ± 0.12 ^a^	0.36 ± 0.02 ^a^	0.38 ± 0.07 ^a^	44/5.73	49/5.79	185	5	4	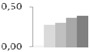
450	enolase	*Arabidopsis* *lyrata subsp.* *lyrata*	gi|297820024	0.26 ± 0.16 ^a^	0.09 ± 0.06 ^a^^,b^	0.03 ± 0.04 ^b^	0.03 ± 0.02 ^b^	0.03 ± 0.03 ^b^	48/5.57	57/5.97	323	10	5	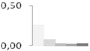
479	fructose-bisphosphate aldolase, cytoplasmic	*Zea mays*	gi|162462282	0.01 ± 0.02 ^b^	0.05 ± 0.05 ^b^	0.25 ± 0.06 ^a^^,b^	0.41 ± 0.08 ^a^	0.37 ± 0.18 ^a^	39/7.52	54/6.62	191	10	4	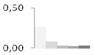
491	isovaleryl-CoA dehydrogenase	*β vulgaris*	gi|112005099	0.16 ± 0.04 ^b^	0.21 ± 0.03 ^b^	0.45 ± 0.09 ^a^^,b^	0.62 ± 0.20 ^a^	0.58 ± 0.15 ^a^	46/6.71	54/6.27	301	13	11	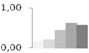
531	cytosolic aldolase	*Fragaria x* *ananassa*	gi|10645188	0.02 ± 0.03 ^c^	0.12 ± 0.02 ^b^^,c^	0.36 ± 0.17 ^a^^,b^	0.48 ± 0.15 ^a^	0.43 ± 0.13 ^a^^,b^	38/6.93	54/6.45	232	16	6	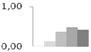
*Genetic information processing*														
14	60S ribosomal protein L22, putative	*Ricinus* *communis*	gi|255551787	0.03 ± 0.05 ^a^	0.09 ± 0.04 ^b^	0.30 ± 0.06 ^c^	0.58 ± 0.16 ^c^^,d^	0.85 ± 0.10 ^d^	14/9.54	19/4.52	279	37	7	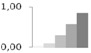
18	40 S Ribosomal protein	*Vitis vinifera*	gi|225465502	0.29 ± 0.05 ^a^	0.20 ± 0.05 ^a^^,b^	0.13 ± 0.12 ^a^^,b^	0.06 ± 0.08 ^b^	0.06 ± 0.08 ^b^	15/5.84	19/5.43	510	34	23	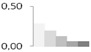
90	proteasome subunit α	*Ricinus* *communis*	gi|255583952	0.01 ± 0.01 ^c^	0.08 ± 0.07 ^b^^,c^	0.12 ± 0.04 ^b^^,c^	0.31 ± 0.09 ^a^	0.24 ± 0.09 ^a^^,b^	28/5.84	30/6.49	179	19	5	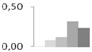
134	glycine-rich RNA-binding protein	*Prunus avium*	gi|34851124	0.04 ± 0.04 ^c^	0.08 ± 0.07 ^c^	0.15 ± 0.06 ^b^^,c^	0.31 ± 0.02 ^a^^,b^	0.36 ± 0.11 ^a^	17/7.82	17/4.87	127	13	3	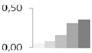
395	P0 ribosomal protein-like	*Solanum* *tuberosum*	gi|78191424	0.33 ± 0.10 ^c^	0.92 ± 0.31 ^b^^,c^	1.11 ± 0.16 ^a^^,b^	1.71 ± 0.37 ^a^	1.36 ± 0.34 ^a^^,b^	33/5.12	43/5.44	200	16	5	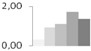
397	P0 ribosomal protein-like	*Solanum* *tuberosum*	gi|78191424	0.43 ± 0.12 ^b^	1.07 ± 0.78 ^a^^,b^	2.38 ± 1.05 ^a^^,b^	1.90 ± 0.93 ^a^^,b^	2.57 ± 0.39 ^a^	33/5.12	43/5.54	313	21	8	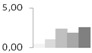
396	60S acidic ribosomal protein P0	*Lupinus* *luteus*	gi|1710585	4.35 ± 0.91 ^b^	6.29 ± 0.81 ^a^^,b^	7.01 ± 1.55 ^a^^,b^	8.62 ± 1.80 ^a^	8.26 ± 1.00 ^a^	34/5.07	42/5.49	91	10	3	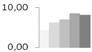
403	26S protease regulatory subunit 6A homolog	*Brassica rapa*	gi|3024434	0.10 ± 0.03 ^c^	0.15 ± 0.02 ^c^	0.25 ± 0.02 ^b^	0.45 ± 0.02 ^a^	0.45 ± 0.05 ^a^	48/4.92	51/5.02	139	10	3	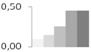
494	putative 60S acidic ribosomal protein P0	*Trifolium* *pratense*	gi|84468360	0.45 ± 0.18 ^c^	0.92 ± 0.23 ^b^^,c^	1.57 ± 0.36 ^a^^,b^	2.22 ± 0.55 ^a^	1.71 ± 0.51 ^a^^,b^	34/5.27	44/5.16	111	12	4	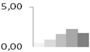
518	proteasome subunit β type-2-A-like	*Vitis vinifera*	gi|359479647	0.03 ± 0.03 ^c^	0.13 ± 0.03 ^b^	0.14 ± 0.03 ^a^^,b^	0.19 ± 0.05 ^a^^,b^	0.23 ± 0.04 ^a^	22/5.85	26/6.64	110	22	5	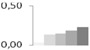
521	60S ribosomal protein L23a	*Nicotiana* *tabacum*	gi|585876	0.31 ± 0.01 ^c^	0.28 ± 0.25 ^c^	0.48 ± 0.07 ^b^^,c^	0.68 ± 0.06 ^a^^,b^	0.84 ± 0.06 ^a^	17/10.18	28/4.98	97	14	2	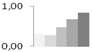
*Cellular processes*														
179	α tubulin	*Plantago* *major*	gi|106879605	0.02 ± 0.03 ^c^	0.07 ± 0.01 ^b^^,c^	0.11 ± 0.04 ^b^	0.20 ± 0.03 ^a^	0.23 ± 0.02 ^a^	30/4.7	29/5.88	131	6	2	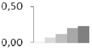
253	charged multivesicular body protein 4b, putative	*Ricinus* *communis*	gi|255546239	0.00 ± 0.01 ^b^	0.01 ± 0.02 ^b^	0.04 ± 0.03 ^b^	0.05 ± 0.01 ^b^	0.24 ± 0.10 ^a^	24/4.80	44/4.98	223	27	5	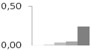
*Unclassified*														
213	type IIIa membrane protein cp-wap13	*Vigna* *unguiculata*	gi|2218152	1.84 ± 0.54 ^a^	2.42 ± 0.42 ^a^	2.46 ± 0.19 ^a^	1.81 ± 0.07 ^a^^,b^	0.73 ± 0.56 ^b^	40/6.24	15/5.43	115	7	2	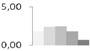
443	late embryogenesis abundant protein D-34-like	*Brachypodium* *distachyon*	gi|357115298	0.12 ± 0.04 ^b^	0.53 ± 0.77 ^a^^,b^	0.72 ± 0.64 ^a^	0.50 ± 0.84 ^a^^,b^	0.03 ± 0.03 ^b^	6/8.23	15/6.03	94	6	1	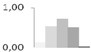
*Unknown*														
434	unknown			8.31 ± 0.83 ^a^^,b^	8.67 ± 0.15 ^a^	6.93 ± 0.76 ^b^^,c^	5.63 ± 0.38 ^c^^,d^	4.90 ± 0.29 ^d^		14/5.27				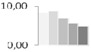
435	unknown	*Ricinus* *communis*	gi|255575865	1.90 ± 0.16 ^a^	2.00 ± 0.14 ^a^	1.23 ± 0.19 ^b^	0.47 ± 0.11 ^c^	0.21 ± 0.03 ^c^	16/10.23	17/5.23	84	19	3	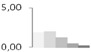
506	unknown			1.99 ± 0.30 ^a^	1.47 ± 0.13 ^a^	0.65 ± 0.04 ^b^	0.24 ± 0.08 ^b^	0.43 ± 0.53 ^b^		15/5.39				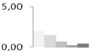

^a^ The spot number is the index in the reference gel; ^b^ The mean value with the standard deviation of three spot volumes at each analyzed stage: 14, 16, 18, 20 and 22 WAF. Levels not connected by the same letter are significantly different; ^c^ Protein sequence coverage; ^d^ Number of peptide sequences.

As listed in Table 1, the spots represented 17 non-redundant proteins. The percentage of sequence coverage ranged from 4% to 69%, and the number of identified peptides varied from one to 175. Among the 17 spots, 11 corresponded to woody plant sequences (four to *Ricinus communis*, three to *Litchi chinensis*, two to *Vitis vinifera*, one each to *Betula pendula* and *Populus trichocarpa*). Homologous proteins were found for 16 of the spots, while one spot was characterized as unknown. The Kyoto Encyclopedia of Genes and Genomes (KEGG) database (Kyoto University, Kyoto, Japan, and University of Tokyo, Tokyo, Japan) [22] was used to provide a functional classification for the identified proteins. They were classified into four categories: metabolism (seven proteins); genetic information processing (three proteins); cellular processes (six proteins); and unclassified (one protein). Based upon the use of a Tukey HSD test, five proteins, including two α- and two β-tubulins (Spots 171, 174, 175 and 183, respectively) and actin (Spot 154) decreased in abundance during the 10–13 WAF stage of morphogenesis. Two proteins, the cytochrome b6-f complex iron-sulfur (Rieske iron-sulfur protein) subunit (Spot 222) and triose phosphate isomerase (Spot 244), exhibited an increase in abundance. The abundance of the other isolated proteins exhibited variable patterns during the 10–13 WAF sampling period.

### 2.2. Proteomic Analysis of Embryo Maturation

Although cotyledons contribute to the depth of dormancy in an embryo, the embryonic axis itself develops a well-defined role in dormancy [23], therefore, the second period of sampling focused specifically on this part of the embryo. Since 14 WAF embryos are morphologically fully formed, further maturation studies were conducted on *A. platanoides* embryo axes collected between 14 and 22 WAF. An increase in the fresh weight of embryo axes was observed from 14 to 16 WAF, after which the fresh weight decreased (Figure 2B). Protein levels in embryo axes increased during the 14 to 18 WAF sampling period and then, afterwards, decreased (Figure 2B).

As with the morphogenetic analysis, 2D gels of separated proteins were used to identify differentially abundant proteins in samples collected during the embryo maturation stage (14–22 WAF). On average, 462 spots were detected on 2D gels. A total of 33 spots, representing 7% of the total number of spots on a master gel (Figure 3B), exhibited significant changes in abundance as determined by ANOVA and Tukey-Kramer HSD and were identified by MS.

The differentially abundant spots represent 33 non-redundant proteins (Table 2). The percentage of sequence coverage ranged from 3% to 37%, and the number of identified peptides varied from one to 27. Among the 33 spots, 16 corresponded to woody plant sequences (six to *V. vinifera*, five to *R. communis* and one each to *Catharanthus roseus*, *Retama raetam*, *Alnus glutinosa*, *Prunus avium* and *Taxus baccata*). Homologous proteins were identified for 30 spots, while three spots were characterized as unknown. Identified proteins were classified into four categories as determined by KEGG analysis: metabolism (15 proteins); genetic information processing (11 proteins); cellular processes (two proteins); and unclassified (two proteins).

Thirty three spots displayed significant variations in abundance during the 14–22 WAF period of embryo maturation (Table 2). Based on the Tukey HSD test, five proteins, including three unknown (Spots 434, 435 and 506), an enolase (450) and a 40S ribosomal protein (Spot 18) exhibited a decrease in abundance during maturation. Most of the proteins (23 spots), however, exhibited an increase in abundance. The abundance of the other identified proteins exhibited variable patterns during the 14–22 WAF sampling period.

### 2.3. Discussion

Seed development is a crucial stage of plant life and has been the topic of a great deal of biological research. In *A. platanoides*, as in many species, seeds acquire dormancy and desiccation tolerance during development to enable them to undergo long periods of storage and remain viable until suitable environmental conditions are present for growth. The present study focused on characterizing the proteome of two stages, morphogenesis and physiological maturation of developing *A. platanoides* seeds in order to identify regulatory components associated with dormancy acquisition, desiccation tolerance and seed growth.

During morphogenesis, the embryo develops its shape and size. Embryos of *A. platanoides* acquired maximum growth at 14 WAF. Analysis of the proteome indicated that proteins associated with metabolism and cellular processes were the most frequently represented during morphogenesis.

At 18 WAF, a decrease in embryo fresh weight was observed, suggesting that programmed desiccation processes had been initiated. Our results confirmed the observation of Pukacka [24], that *A. platanoides* seeds attain desiccation tolerance after the acquisition of maximum reserves, as reflected by maximum fresh and dry weight.

*A. platanoides* seeds acquire dormancy during development. Pinfield *et al.* [23] reported that excised immature embryos of *A. platanoides* seeds, isolated from very young developing fruits, had high levels of germination, but that the ability to germinate decreased rapidly as development proceeded, reaching minimum values by 18 WAF and remaining low until maturity. Results of our previous study demonstrated that the most intensive qualitative and quantitative changes in protein content occur at the end of seed maturation [14]. In the present study, our analysis of the proteome indicated that the majority of differentially abundant proteins were observed during seed maturation and were involved in metabolism pathways and genetic information processing.

The proteins identified during seed development in the present study display a wide range of biochemical activities, based on their functional classification in the KEGG database [22]. Based on metabolomic, proteomic and genomic data, the involvement of these proteins in developmental processes, with an emphasis on dormancy acquisition, will be discussed in relation to their function.

#### 2.3.1. Defense Proteins

Reactive oxygen species (ROS) play a role in cellular signaling and are an inherent part of metabolic processes, such as respiration/photosynthesis and stress response [25]. They have been reported to play a key role in the shift from a dormant to a non-dormant state in seeds [26]. Bailly *et al.* [27] suggested that the ability of seeds to germinate depends on the accumulation of a critical level of H_2_O_2_. In the present study, an increase in antioxidant proteins (which are involved in the regulation of the ROS level) was observed, during both morphogenesis (Cu/Zn superoxide dismutase (SOD), Spot 8) and physiological maturation (glutathione S-transferase (GST), 32; ascorbate peroxidase (APX), 400; and monodehydroascorbate reductase (MDAR), 189).

Our results showed that the highest levels of APX occurred two weeks after the desiccation and dormancy processes had been initiated. This result confirms earlier data obtained by Pukacka and Ratajczak [28]. These authors suggested that APX plays an important role in the acquisition of tolerance to desiccation, the protection from ROS and in protein maturation. Lee *et al.* [29] reported that overexpression of *APX* and Cu/Zn *SOD* genes in transgenic tobacco seeds did not have a negative impact on seed development and that the transgenic seeds exhibited enhanced longevity and germination rates under stress conditions. The data suggest that the role of APX in seed development can be both regulatory and protective.

GSTs are multifunctional proteins involved in the conjugation of the reduced form of glutathione (GSH) to substrates for the purpose of detoxification [30]. Although GSTs are thought to play a major role in oxidative stress metabolism, little is known about the regulatory function of GSTs [31]. In plants, GST synthesis is induced by phytohormones, such as ethylene, cytokinin, auxin, abscisic acid and salicylic acid [32]. In *Arabidopsis*, Chen *et al.* [31] reported that AtGSTU17 participates in light signaling and may regulate various aspects of development by modulating GSH pools in coordination with phytochrome. Mutant *atgstu17* plants accumulated higher levels of GSH and abscisic acid (ABA), exhibited hyposensitivity to ABA during seed germination and better development of primary and lateral root systems [31]. These data indicate that the relationship between GST and ABA, a hormone that controls dormancy, can play a significant role in the regulation of seed development.

Two other proteins isolated from developing Norway maple seeds are also involved in the antioxidative pathway. The abundance of both of these enzymes continually increased during seed maturation. Methionine sulfoxide reductase (MSR, Spot 242) catalyzes the regeneration of methionine sulfoxides (products of oxidation) to methionine and thioredoxin [33]. Besides scavenging oxidative species as part of a detoxification process, methionine modification can also result in protein unfolding, resulting in the modification of the biological function of the affected protein [33]. This data suggest that besides a protective role, MSR may regulate the activity of the other proteins, including those regulating seeds development, by their modification. Glyoxalase I (lactoylglutathione lyase, Spot 391) is involved in tolerance to oxidative stress, because of its ability to facilitate the detoxification of methylglyoxal produced during glycolysis and photosynthesis [34]. The activity of this enzyme is stimulated by several factors, including cell growth, hormones and stress conditions [35]. Transgenic tobacco modified to reduce glyoxalase I gene expression showed enhanced accumulation of methylglyoxal, which resulted in the inhibition of seed germination [35]. The function of glyoxalase I in seed development is associated with protection against the toxicity of methylglyoxal.

#### 2.3.2. Metabolic Pathways Activated during Seed Development

The establishment of new cells and tissues during embryogenesis requires carbon skeletons for the synthesis of new compounds and energy for biochemical reactions. The present study of embryogenesis identified many proteins associated with carbohydrate and energy metabolism. The differential abundance of enzymes of the glycolysis/gluconeogenesis pathway was detected, including triosephosphate isomerase (27, 244, 222), enolase (402, 407, 450) and glyceraldehyde-3-phosphate dehydrogenase (263). A developmental associated pattern of protein abundance was also observed for an enzyme in the Calvin cycle, fructose-bisphosphate aldolase (252, 479, 531). A protein related to photosynthesis, oxygen evolving enhancer protein (119), also fluctuated in abundance. Triosephosphate isomerase (TPI) plays an important role in carbon metabolism, catalyzing the reversible conversion of the glycolytic intermediates, dihydroxyacetone phosphate (DHAP) and glyceraldehyde-3-phosphate (GAP). DHAP is a precursor for the glycerol moiety in glycerolipids; GAP and/or DHAP are also precursors for a number of secondary metabolites [36]. Dorion *et al.* [37] demonstrated that a large reduction of cytosolic TPI alters the distribution of carbon in plant primary metabolism. In our study, the abundance of TPIs increased as seed development progressed, most likely due to the demand for carbon skeletons during the growth of embryo tissues.

The abundance of fructose-bisphosphate aldolase (252) decreased during the latter stage of morphogenesis; however, the abundance of two other aldolases (479, 531) increased during maturation, reaching a maximum level at 20 WAF. ABA delayed the completion of dormancy and inhibited the germination of *A. platanoides* seeds by decreasing the abundance of aldolase [9]. Gibberellins (GA), antagonists of ABA, increased the abundance of aldolase in rice roots [38]. Aldolase was physically associated with vacuolar H-ATPase, and the authors suggested that it may regulate the cell elongation that determines root length [38]. The role of aldolase in seed development may be associated with the demand for energy and the growth of the embryo.

The abundance of three enolases (402, 407, 450) changed during physiological maturation of the embryo. Spot 402 and 407 increased in abundance, while 450 decreased. The abundance of glyceraldehyde-3-phosphate dehydrogenase (263) also increased during maturation. These glycolysis/gluconeogenesis enzymes are involved in supplying energy and the synthesis of carbon skeletons.

The abundance of the oxygen evolving enhancer (OEE) protein I (119) was variable during morphogenesis (Table 1). In higher plant species, OEEs are involved in the water-splitting reaction, which is part of the photosynthetic oxygen-evolving process in photosystem II (PSII) [39]. OEE has been associated with stress adaptation [40]. The presence of OEE1 during seed morphogenesis can be explained by the fact that the photosynthetic system is established and functional in embryos of *A. platanoides*, while seeds are still attached to the mother plant (whole seeds are green because of the chlorophyll).

Succinate semialdehyde dehydrogenase (SSADH, 401) is an enzyme involved in the γ-aminobutyrate (GABA) shunt pathway. SSADH catalyzes the conversion of succinic semialdehyde (SSA) to succinate [41]. The GABA shunt is a conserved metabolic pathway found in bacteria, animals and plants that bypasses the tricarboxylic acid cycle [42]. Toyokura *et al.* [41] suggested that a GABA shunt metabolite, SSA or a close derivative, is involved in leaf development. Considerable amounts of GABA are formed in germinating seeds. A study of SSADH metabolism indicated that the mobilization of respiratory substrates during *R. communis* seed germination involves the GABA shunt pathway [43]. It is likely that this is the same role that SSADH plays during seed development in *A. platanoides*.

Isovaleryl-CoA dehydrogenase (491) is an enzyme associated with the catabolism of lysine. Although the products of amino acid catabolism are generally channeled into the tricarboxylic acid cycle, it has also been demonstrated that isovaleryl-CoA dehydrogenase provides electrons to the ubiquinol pool in plant cells via the flavoprotein/electron transfer flavoprotein:ubiquinone oxidoreductase (ETF-ETFQO complex) [44,45]. Moreover, the results observed under a range of different growth conditions suggest a role for the ETF-ETFQO pathway, not only during stress, but also under different conditions experienced by most plants at some stage during their lifecycle [44,45].

Proline iminopeptidase (PAP, 408), whose abundance increased during embryo maturation, is an enzyme that specifically removes *N*-terminal proline from peptides [46]. The action of PAPs can be of biological significance, leading to the inactivation or biotransformation of peptides and proteins. Concomitant expression of genes for proline synthesis and proline degradation has been repeatedly demonstrated in *Arabidopsis*, raising questions about the role of proline cycling in seed development [47]. Continued studies investigating the role of PAPs and their target, proline, will provide additional information on their function in seed development.

#### 2.3.3. Unclassified Proteins

#### Cell Wall Biosynthesis

The Golgi apparatus-associated type IIIa membrane protein, cp-wap13 (213), whose abundance first increased, then decreased during maturation, is involved in cell wall polysaccharide (cellulose) biosynthesis [48]. The level of this protein increased when the process of bud dormancy release was initiated in *Pinus sylvestris*, suggesting that it may be involved in this process [49]. During the physiological maturation of *A. platanoides* seeds, this protein reached the highest value at 18 WAF, when dormancy is initiated, suggesting that the level of membrane protein cp-wap13 is probably due to the onset of dormancy. It is likely that also desiccation can decrease the level of this protein at the end of maturation.

#### Late Embryogenesis Abundant (LEA)

In the present study, the abundance of the LEA protein, D-34-like (443) protein, was the highest at 18 WAF. In plants, members of the family of hydrophilic LEA proteins accumulate to high levels during the last stage of seed maturation and are believed to play a protective role during episodes of desiccation stress [50]. Most genes encoding LEA proteins have abscisic acid responsive (ABRE) and/or low temperature responsive (LTRE) regulatory elements in their promoters and are induced by abscisic acid, cold or drought [51]. Three dehydrins (group 2 LEA proteins) in seeds of three *Acer* species, including *A. platanoides*, exhibited a classic response to dehydrative stress and are presumed to ameliorate the injury associated with desiccation [52]. Xu *et al.* [53] identified eight LEA proteins, including LEA D-34, in developing rice embryos. LEA D-34 belongs to group 5 LEA proteins, whose corresponding transcripts accumulate during the late stage of seed development and in response to drought [54]. These proteins are neither enzymes nor typical storage proteins, but probably serve to protect embryos from desiccation stress by stabilizing other proteins and membranes, thus protecting the cell from injury [55]. The role of LEA D-34 in *A. platanoides* seeds seems to be related to desiccation tolerance rather than dormancy acquisition.

#### Cellular Processes

The abundance of an actin (154) and α- (171, 174) and β- (175, 183) tubulins decreased during morphogenesis. During maturation, an increase in the abundance of α-tubulin (179) was observed. The plant cell cytoskeleton is composed of actin filaments, microtubules and associated proteins, which stabilize cytoplasmic structure, anchor proteins, regulate cytoplasmic streaming and participate in the assembly of the mitotic spindle and construction of the cell wall [56]. Microtubules are heterodimeric polymers of α/β-tubulin, encoded by multiple genes. The development of grape (*V. vinifera*) primordia into leaves, tendrils or flowers was associated with differences in the accumulation of specific tubulin isoforms required for the assembly of microtubule arrays with distinct functions [57]. Previous research demonstrated that changes in the abundance of α- and β-tubulins were associated with dormancy breaking and germination of *A. platanoides* seeds [58]. The present study indicates that tubulins are also associated with seed embryogenesis, conceivably with the growth of embryo, as well as the process of dormancy acquisition.

The abundance of charged multivesicular body protein 4b (CHMP4B) was highest in mature embryos at 22 WAF. CHMP4B is a subunit of an endosomal sorting complex, which is required for transport (ESCRT) and is essential to cytokinesis [59]. ESCRT is involved in the abscission of the narrow membrane bridge connecting two daughter cells in the final stage of cytokinesis [59]. Most embryonic nuclei of dry, fully-mature *A. platanoides* seeds are arrested in the G_2_ phase of the cell cycle [58]. Therefore, it seems that during physiological maturation of the embryo, when CHMP4B is accumulating, cells are preparing for the cytokinesis that will occur when dormancy is complete and the seed germinates.

#### Genetic Information Processing

During the maturation phase of embryo development, an increase in the abundance of almost all of the identified ribosomal proteins (besides 60S ribosomal protein, L22, Spot 18) was observed, such as 60S acidic ribosomal protein P0 (494, 395, 396, 397), 60S ribosomal protein L23 (521) and L22 (14). This may reflect an increase in protein translation activity during seed maturation.

The glycine-rich RNA-binding protein (GRRBP, 134) also increased in abundance during embryo maturation. GRRBP has been implicated in post-transcriptional regulation of gene expression in plants under various stress conditions. The synthesis of an ABA-responsive glycine-rich protein was correlated with the level of seed dormancy in beech (*Fagus sylvatica*) seeds [60]. Mortensen *et al.* [61] later reported that the degree of dormancy of beech seeds is associated with the expression of the dormancy-related gene, *GRPF1*, encoding a GRRBP. ABA, which inhibited the germination of *A. platanoides* seeds, also increased the abundance of GRRBP [9]. Collectively, the data indicate that GRRBP in seeds plays a role in dormancy acquisition by post-transcriptional regulation of gene expression.

Proteasome proteins represented by 26S protease regulatory subunit 6A (403), proteasome subunit α (90) and proteasome subunit β type-2-A-like (518) increased in abundance during the maturation phase of embryo development. In plants, the ubiquitin-proteasome system is associated with the regulation of developmental events in proliferating cells and developing tissues by controlling the level of nuclear regulatory proteins (transcription factors) [62]. Molecular studies in *Arabidopsis* have provided evidence for the role of a ubiquitin/26S proteasome system in ABA responses [63]. ABA is the main hormone responsible for physiological seed dormancy initiation and maintenance [2]. Proteasomes can be involved in the acquisition of seed dormancy by their ability to degrade transcriptional regulators that effect diverse metabolic pathways, including ABA signal transduction.

The nonspecific lipid transfer protein (nsLTP, 1) exhibited variable abundance during the morphogenesis of *A. platanoides* embryos. nsLTPs are a group of abundantly expressed, small, basic proteins that can reversibly bind and transport hydrophobic molecules. They have been reported to play a role in a variety of plant processes, including wax synthesis and transport, abiotic stress resistance, disease resistance and plant reproduction [64]. They are induced by ABA [65], and their association with embryogenesis was also observed by Sterk *et al.* [66] and Thoma *et al.* [67].

## 3. Experimental Section

### 3.1. Plant Material and Experimental Design

Seeds were collected from one tree of Norway maple (*Acer platanoides* L.) located in the Kórnik Arboretum (Poland, N52°14' and E17°05'). Weekly collections (3 × 30 seeds) were made between 10 and 22 weeks after flowering (WAF), which occurred between 1 July and 29 September 2011. Because the monitoring of a group of trees showed that flowering started (and ended) at different times, just one tree was chosen for all of the experiments. The tree for the experiments ended flowering on 22 April 2011. The collection of samples was designed in two parts: (1) intact embryos were collected between 10 and 13 WAF, the period corresponding with morphogenesis; and (2) embryo axes were collected between 14 and 22 WAF, the period corresponding with physiological maturation. Material was immediately frozen in liquid nitrogen and then stored at −80 °C until further processing.

The experiment was designed separately for morphogenesis and for maturation. Since mainly the embryonic axis itself develops a well-defined role in dormancy [23], therefore, the second part of experiment focused specifically on this part of the embryo. Another reason was also that we would not like to concentrate the research on storage proteins present in cotyledons.

### 3.2. Protein Extraction

Proteins were extracted and precipitated overnight at −20 °C in a 10% (*w*/*v*) solution of TCA in acetone containing 20 mM dithiothreitol (DTT) [9]. After centrifugation (16,000× *g* for 5 min at 4 °C), the resulting pellets were washed three times with 1 mL of acetone supplemented with 20 mM DTT and were then re-centrifuged. After vacuum drying, pellets were resuspended in lysis buffer (7 M urea, 2 M thiourea, 2% (*w*/*v*) 3-[(3-cholamidopropyl)dimethylammonio]-2-hydroxy-1-propanesulfonate (CHAPS), 1.5% (*w*/*v*) DTT, 0.5% (*v*/*v*) IPG buffer pH 4–7), supplemented with the Protease Inhibitor Cocktail (Roche, Basel, Switzerland), according to the manufacturer’s suggestions. Protein concentrations were determined using the Bradford assay [68].

### 3.3. Protein Electrophoresis, 2-DE SDS-PAGE

All analyses were conducted using three biological replicates (3 × 30 seeds) from the same tree. Proteins (600 mg for colloidal Coomassie Blue) were first separated according to their charge on rehydrated Immobiline dry strips (24 cm, with a linear pH gradient of 4–7) in rehydration buffer (6 M urea, 2 M thiourea, 2% (*w*/*v*) CHAPS, 20 mM (*w*/*v*) DTT, 0.5% (*v*/*v*) IPG buffer pH 4–7) using an Ettan IPGphor 3 IEF System (GE Healthcare Life Science, Uppsala, Sweden). The electrophoresis program used for isoelectro-focusing was according to the manufacturer’s directions for 24-cm strips. The strips containing the separated proteins were either stored at −80 °C or directly immersed in equilibration Solution I (6 M urea, 1.5 M Tris-HCl, pH 8.8, 30% (*v*/*v*) glycerol, 2% (*w*/*v*) SDS, 1% (*w*/*v*) DTT) for 10 min and then for the same length of time in equilibration Solution II (equilibration Solution I supplemented with 2.5% (*w*/*v*) iodoacetamide, without DTT) and subjected to second dimension (SDS-PAGE) separation.

Pre-cast Ettan DALT 12.5% (*w*/*v*) polyacrylamide gels (GE Healthcare Life Science) and the Ettan DALT Six electrophoresis chamber (capable of processing six gels) were used for SDS-PAGE. Conditions were as follows: 1 h at 80 V and 5 h at 500 V. A mixture of molecular weight markers (GE Healthcare) was loaded next to the Immobiline strip. Triplicate gels were run for every sample (*n* = 3). After electrophoresis, the gels were stained with colloidal Coomassie Blue, which, in addition to visualization and quantification, also allowed for downstream MS analyses [69].

### 3.4. Gel Analysis

The gels were scanned and analyzed using 2D Image Master 7 Platinum software (GE Healthcare). After spot detection, gels from three independent biological samples were aligned, and normalized spot volumes of the identified spots were determined. Spots with variations in abundance (statistics between gel sets/classes, *i.e.*, central tendency, dispersion and overlapping measures, was used) were subjected to ANOVA and the Tukey-Kramer HSD test (SAS Institute) to select spots that significantly varied (*p* < 0.05) in abundance during morphogenesis and physiological maturation. The significantly variable proteins were subsequently identified by MS.

### 3.5. Mass Spectrometry (MS)

The gel spots were subjected to a standard “in-gel digestion” procedure in which proteins were reduced with 10 mM (*w*/*v*) DTT (for 30 min at 56 °C), alkylated with 55 mM iodoacetamide (45 min in the dark at room temperature) and digested overnight with trypsin (Sequencing Grade Modified Trypsin, Promega V5111, Promega, Madison, WI, USA) in 25 mM ammonium bicarbonate (25 ng·µL^−1^ of trypsin). The resulted peptides were eluted from the gel matrix with 0.1% (*v*/*v*) trifluoroacetic acid (TFA) in 2% (*v*/*v*) Acetonitrile (can).

Peptide mixtures were analyzed by liquid chromatography coupled to the mass spectrometer in the Laboratory of Mass Spectrometry, Institute of Biochemistry and Biophysics, the Polish Academy of Sciences (Warsaw, Poland). Samples were concentrated and desalted on an RP-C18 pre-column (nanoACQUITY Symmetry^®^ C18, Waters, Milford, MA, USA), and further peptide separation was achieved on a nano-Ultra Performance Liquid Chromatography (UPLC) RP-C18 column (Waters, BEH130 C18 column, 75 µm i.d., 250 mm long) of a nanoACQUITY UPLC system, using a linear acetonitrile gradient (0%–60% (*v*/*v*) ACN for 120 min) in the presence of 0.05% (*v*/*v*) formic acid with a flow rate of 150 nL/min. The column outlet was directly coupled to the electrospray ionization (ESI) ion source of the Orbitrap Velos type mass spectrometer (Thermo Electron Corp., San Jose, CA, USA), working in the regime of the data-dependent MS to MS/MS switch. An electrospray voltage of 1.5 kV was used. A blank run ensuring the lack of cross-contamination from previous samples preceded each analysis.

Acquired raw data were pre-processed with Mascot Distiller software (version 2.3.2.0, Matrix Science, London, UK) followed by a database search using the Mascot Search engine (Mascot Daemon v. 2.3.0, Mascot Server v. 2.2.03, Matrix Science, London, UK) against the NCBInr database (version 20120224) with a *Viridiplantae* filter (884,942 sequences). The search parameters for precursor and product ions mass tolerance were 40 ppm and 0.6 Da, respectively, with an allowance made for one missed trypsin cleavage, and the following fixed modifications, cysteine carbamidomethylation, and allowed variable modifications, lysine carbamidomethylation and methionine oxidation. Protein identification was performed using the Mascot search probability-based molecular weight search (MOWSE) score. The ion score was −10 × log(*P*), where P was the probability that the observed match was a random event*.* Peptides with a Mascot Score exceeding the threshold value corresponding to a <5% false positive rate, calculated by the Mascot procedure, were considered to be positively identified.

## 4. Conclusions

The development of the deep embryo dormancy present in fresh seeds of *A. platanoides* is a progressive process starting from a level of high germination in embryos early in seed ontogeny to one of low germination potential as the embryo approaches maturity [23]. The comparative analysis conducted in the present study identified proteins in Norway maple embryos that changed in abundance during morphogenesis (10–13 WAF) and embryo maturation (14–22 WAF). A total of 17 proteins were identified that changed in abundance during the period of morphogenesis. Five proteins, including two α- and two β-tubulins and an actin were downregulated, while two proteins, a Rieske iron-sulfur protein and a triosephosphate isomerase, were significantly upregulated. The abundance of the other identified proteins exhibited variable patterns of abundance. A total of 33 proteins were identified that changed in abundance during the period of embryo maturation. Results indicated that the greatest changes in protein abundance occurred at 22 WAF when seeds and the embryos within become fully mature. Five proteins, including an enolase, a 40S ribosomal protein and three unknown proteins, became less abundant during maturation. Twenty-three of the proteins, however, increased in abundance, while the abundance of the other identified proteins exhibited a variable pattern of abundance.

Proteomic identification and biological functional analysis of the identified proteins indicated that oxidative stress, carbohydrate and energy metabolism, metabolism of amino acids, cofactors and vitamins, secondary metabolism, genetic information processing and cellular processes were associated with the developmental changes that occur during embryo morphogenesis and maturation. Embryo morphogenesis was characterized by changes in the abundance of proteins (especially tubulins and actin) associated with the growth of the embryo. The acquisition of embryo dormancy occurs during the latter stages of embryo maturation. Embryo maturation was characterized by changes in the abundance of proteins associated with genetic information processing, energy metabolism energy, cellular and antioxidant processes. A glycine-rich RNA-binding protein and proteasome proteins may be directly involved with dormancy acquisition, but this needs to be verified in future experiments.

## Supplementary Files

Supplementary File 1 Supplementary File (XLS, 92 KB)
